# Gelation and Structural Formation of Amylose by In Situ Neutralization as Observed by Small-Angle X-ray Scattering

**DOI:** 10.3390/gels4030057

**Published:** 2018-06-26

**Authors:** Kyoko Yamamoto, Shiho Suzuki, Shinichi Kitamura, Yoshiaki Yuguchi

**Affiliations:** 1 Graduate School of Engineering, Osaka Electro-Communication University, 18-8, Hatsu-cho, Neyagawa, Osaka 572-8530, Japan; de17a001@oecu.jp; 2 Graduate School of Life and Environmental Sciences, Osaka Prefecture University, 1-1 Gakuen-cho, Naka-ku, Sakai, Osaka 599-8531, Japan; shihoo@bioinfo.osakafu-u.ac.jp (S.S.); skita@bioinfo.osakafu-u.ac.jp (S.K.)

**Keywords:** amylose, in situ neutralization, gelation, small-angle X-ray scattering

## Abstract

The gelation and structural formation of two types of amylose in alkaline solution by in situ neutralization was monitored with time-resolved small-angle X-ray scattering (tr-SAXS). Sharp increases of SAXS profile in lower angle region were observed after gelation. The results showed that aggregation of amylose chains led to a gel point with crystal growth. The aggregation appeared to function as a junction zone, and the aggregate structure depended on the molecular weight of amylose. A high-molecular-weight sample was fitted using a Debye-Bueche function, and a low-molecular-weight sample was fitted using a stretched exponential function.

## 1. Introduction

Amylose, a linear polymer of α-1,4 linked d-glucose, is a component of starch in polysaccharides. The other component, amylopectin, is a branched structure that contains α-1,6 linkages. Starch is produced in plants and functions as an energetic material and is a staple food for people. For that reason, its physical properties are important in food science. Determination of the nano-scale structure and the molecular structure is important not only to understand the properties of starch but also to develop new applications for food products.

Starch has a crystal structure composed of a double helix and/or a single helix structure of amylose chains. When an aqueous dispersion of starch is heated, its transparency increases due to gelatinization. The starch granule is broken, increasing the viscosity of the dispersion because the amylose chains form a coil-like conformation upon hydration due to cleavage of hydrogen bonds in the crystal structure. Leaving the dispersion in this condition at room temperature will produce a white turbid gel due to regeneration of hydrogen bonding and recrystallization. Thus, the mechanism of starch gelation can be considered the formation of a cross-linking zone caused by crystallization and aggregation of starch chains. The small-angle X-ray scattering (SAXS) method is suitable for observation of nano-order structure in starch gels [1].

The present study involved an investigation of the mechanism of amylose gelation starting from a solution of completely dissolved amylose. Amylose dissolves in alkaline aqueous solutions due to breakage of the association among chains. In addition, amylose can produce gels from homogeneous solutions through structural formation control using an in situ neutralization method [2]. The structural formation was observed using time-resolved small-angle X-ray scattering (tr-SAXS), which also allowed accurate measurements to determine the dynamics of amylose gelation.

## 2. Results and Discussion

**Figure 1:**
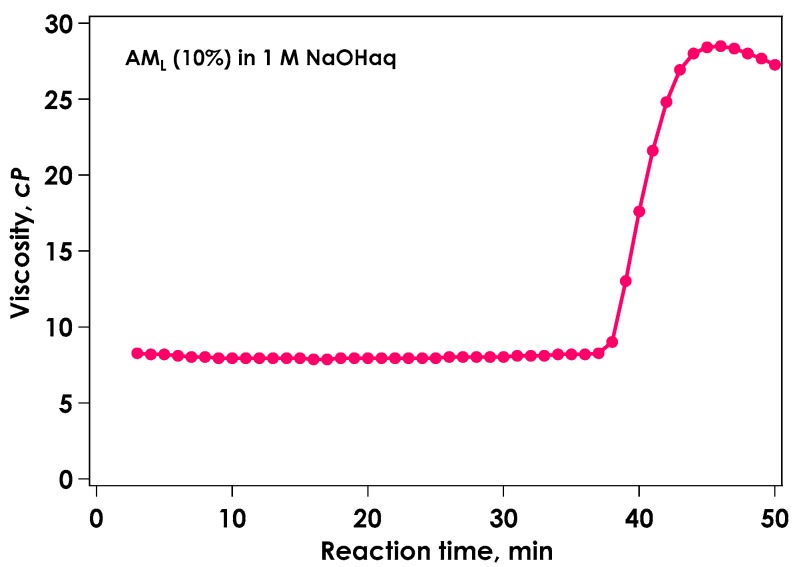
Viscosity behavior at 20 °C during AM_L_ gelation by in situ neutralization.

Amylose dissolves to form a clear liquid in alkaline solutions. Two amylose samples with different molecular weights were used, one with a low molecular weight (*M*_w_ = 1.8 × 10^4^), referred to as AM_L_, and one with a high molecular weight (*M*_w_ = 2.2 × 10^5^), designated as AM_H_. Addition of formamide to amylose alkaline solutions slowly resulted in the formation of a turbid crumbly gel as the pH decreased (in situ neutralization). This process can produce a gel from homogeneous solution. Figure 1 shows the time course of viscosity determined using a rotational viscometer during in situ neutralization of 10% AM_L_ in 1 mol/L aqueous sodium hydroxide upon addition of two equivalents of formamide. Viscosity dramatically increased at the gelation point at 37 min. For this system, the microstructural transformation was observed using time-resolved small-angle X-ray scattering.

**Figure 2:**
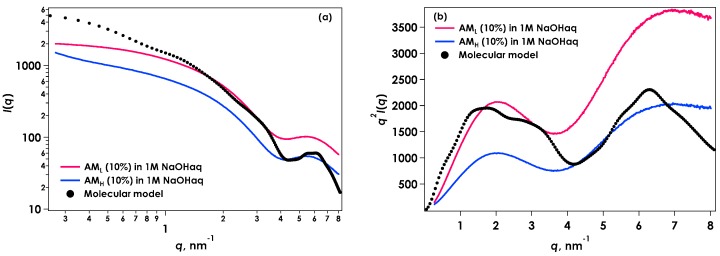
(**a**) Small-angle X-ray scattering (SAXS) from AM_L_ and AM_H_ in 1 M NaOH aq. The scattering curve calculated from the molecular model is also shown; (**b**) Kratky plots [*q*^2^*I*(*q*) vs. *q*] for SAXS data.

Initially, SAXS was used to confirm the molecular chain structure of amylose in the alkaline solution. Figure 2a shows SAXS [*I*(*q*) vs. *q*] results for AM_L_ and AM_H_ in 1 mol/L aqueous sodium hydroxide. *I*(*q*) is the scattering intensity and *q* is the magnitude of the scattering vector defined by (4π/*λ*)sin*θ*, where *λ* is the wavelength of the incident X-rays and 2*θ* is the scattering angle. Only the shapes of the SAXS profiles should be noted, because these scattering intensities have arbitrary units. The SAXS profile in the smaller *q* region from AM_H_ showed a steeper increase in slope than that of amylose AM_L_ due to the higher molecular weight of AM_H_. A Kratky plot [*q*^2^*I*(*q*) vs. *q*] was obtained to produce a characteristic scattering pattern for each type of amylose, as shown in Figure 2b. Both plots showed a maximum near *q* = 2, while other maxima were observed in the larger *q* region [3].

**Figure 3:**
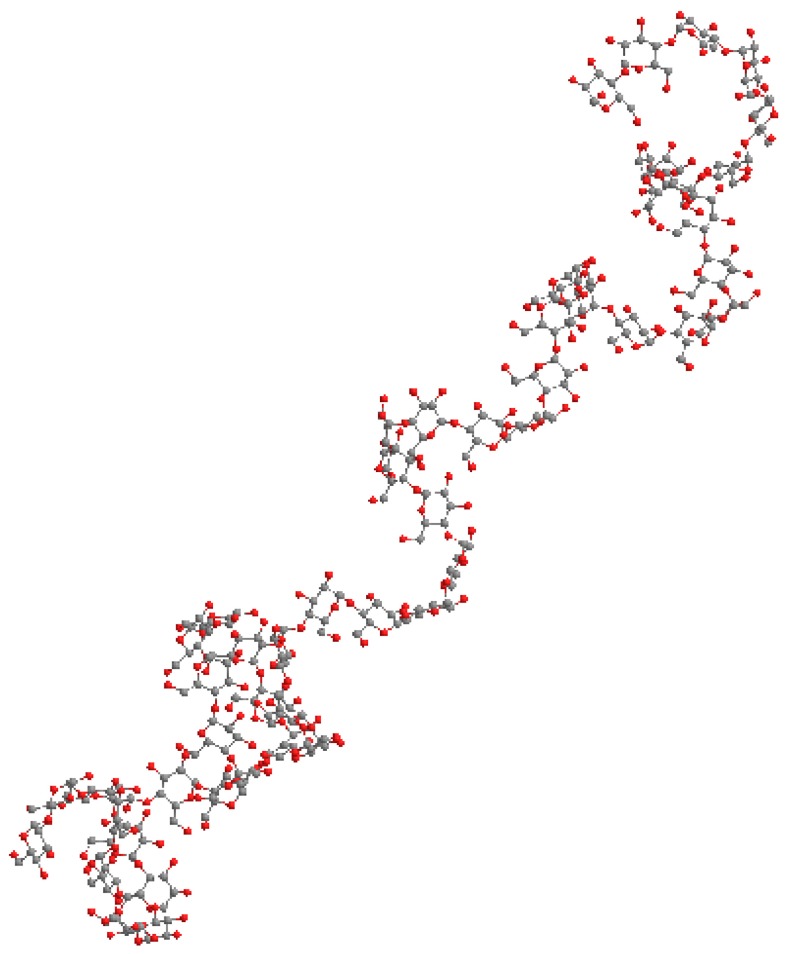
Molecular model of simulated amylose chains.

Figure 3 shows the molecular model of amylose chain (degree of polymerization = 50) conformation simulated using a Monte Carlo method as considering energy map of maltose conformation. Monte Carlo amylosic chains were generated to be distributed consistent with the potential energy of nonbonded nearest-neighbor interactions [4,5,6]. Amylose in the solution state can adopt a helical structure, even with molecular motion. The scattering curve shown in Figure 2 was calculated from this molecular model using the Debye formula [7] by assuming that each atom was sphere with van der Waals radius [8]. Two maxima were present in the Kratky plot, similar to that seen in the experimental data, suggesting that this behavior was characteristic of the helical structure. It is confirmed that the calculated curve can explain experimental data qualitatively. Their deviation may be due to the shortness of model chain and the calculation from only one simulated molecular model. Bayer et al. [9] evaluate that the statistical chain segment of amylose is one turn of the helix. This estimation is correlated with the present molecular model and SAXS results, suggesting partially helical conformation.

**Figure 4:**
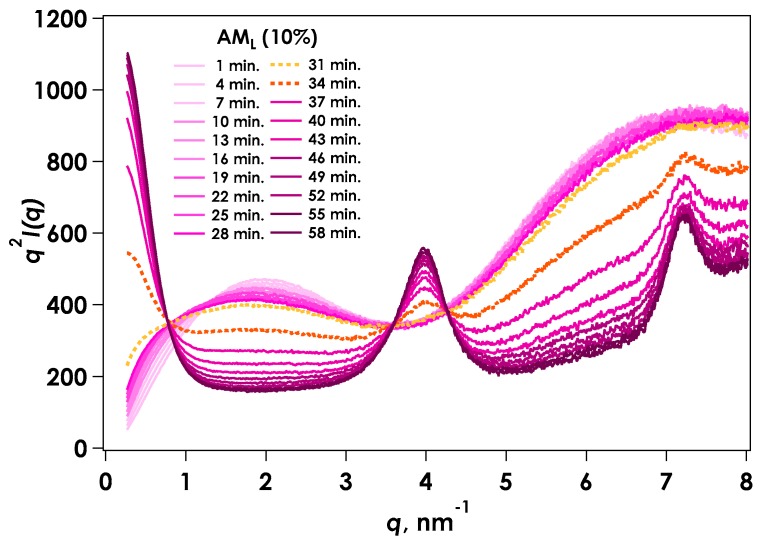
Time variation of Kratky plots for time-resolved SAXS for gelation of AM_L_ by in situ neutralization.

**Figure 5:**
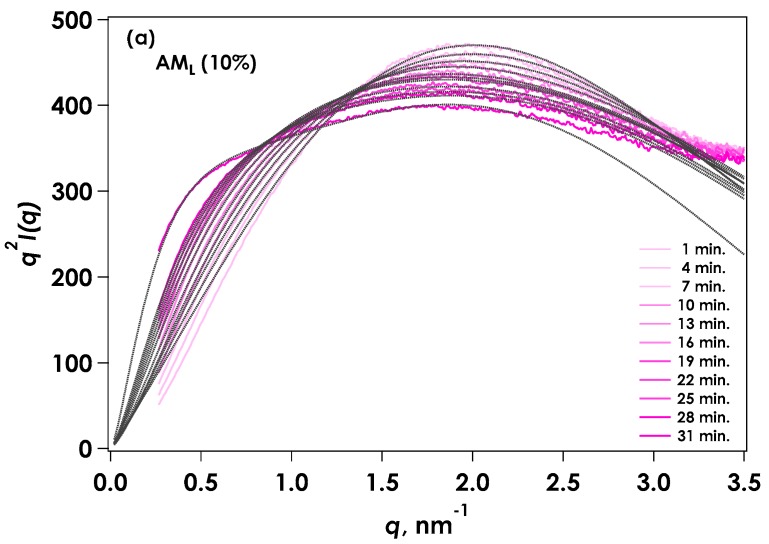

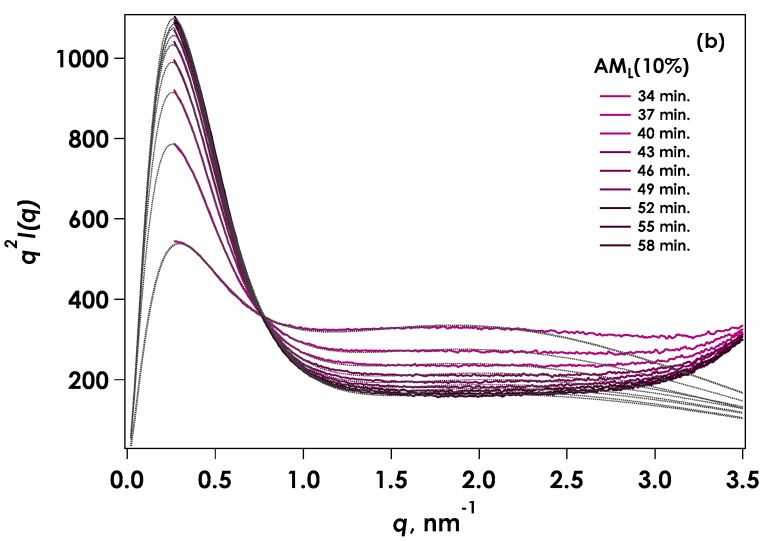
Kratky plots [*q*^2^*I*(*q*) vs. *q*] for SAXS from AM_L_. Dotted lines are fitted curves calculated from Equation (1). (**a**) Initial stage of reaction (1–31 min); (**b**) Final stage of reaction (34–58 min).

Figure 4 shows the Kratky representation for time-resolved SAXS (tr-SAXS) during the gelation process of AM_L_ under the same conditions as those for the viscosity measurement. The scattering intensity around the smaller *q* region (0.1 to 0.8 nm^−1^) increased as the reaction proceeded, indicating aggregation of the molecular chains. The intensity around the middle range of *q* (1 to 3 nm^−1^) tended to decrease. The tr-SAXS showed a steep slope during gelation of AM_L_ from 31 to 35 min, although the gelation point occurred at approximately 37 min according to viscosity measurements. However, the gelation point for AM_H_ occurred at around 31 to 34 min. This SAXS behavior indicates that aggregation of the molecules increased dramatically at the apparent gelation point. At the same time, diffraction peaks were observed at 4 and 7 in the larger *q* range due to the formation of amylose crystals, which suggested that gelation occurred due to amylose crystal growth. The two-component model has been applied for the scattering behavior of chemically crosslinked gels [10,11,12,13,14,15]. It is reported that the amylose gels are composed of two-phase of polymer-rich and water-rich regions by the neutron-scattering method [16]. The present amylose gelation system was similarly composed of a dissolved component and a solid component (corresponding to the aggregation region). This type of excess scattering can be considered as the linear sum of the aggregates, while the soluble portion can be used as a Lorenz-type function for scattering from the polymer chain. The amylose chain is thought to assume a rod-like structure, as observed by SAXS; therefore, a scattering function for a cylindrical shape with a cross-sectional radius (*R_c_*) is more suitable. The aggregated component is considered as a stretched exponential function or Debye-Bueche function [17]. The scattering function of the linear sum of the rod-like particles [18] and the stretched exponential function was applied to the AM_L_ system:

(1) $$I\left(q\right)\sim \frac{1}{q}\cdot {\left[\frac{J_{1}\left(qR_{c}\right)}{qR_{c}}\right]}^{2}+I{\left(0\right)}_{ex}exp\left[-{\left(q\Xi \right)}^{x}\right]$$

where *Ξ* is the mean size of the solid-like non-uniformity and *x* is a positive constant. The results of fitting are displayed in Kratky plots (Figure 5). The calculated results agreed well with the experimental data over the reaction period. In this experiment, the concentration of amylose was set at 10% to ensure the formation of gel. The aggregated region was thought to be highly concentrated, so the dilute region was also appeared. In this case, the diffraction peak found in smaller *q* region from lamellar was not observed. Thus, such kinds of structures as lamellar could not be formed at present concentration range.

**Figure 6:**
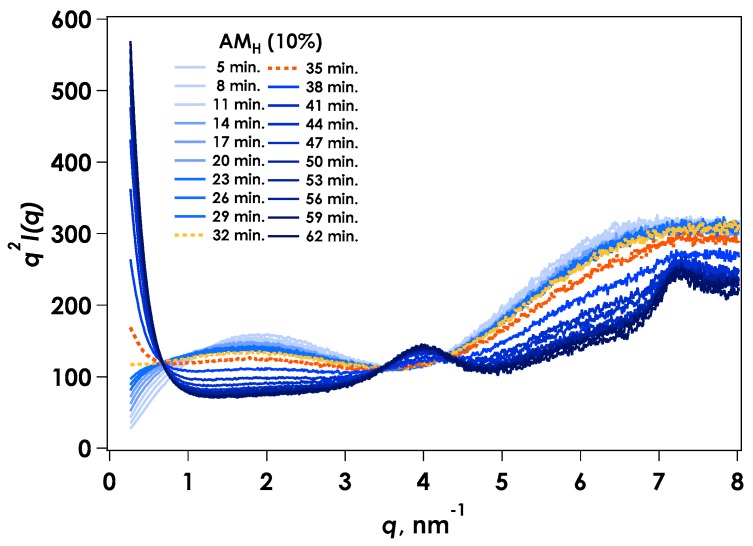
Time variation of Kratky plots for time-resolved SAXS for gelation of AM_H_ by in situ neutralization.

**Figure 7:**
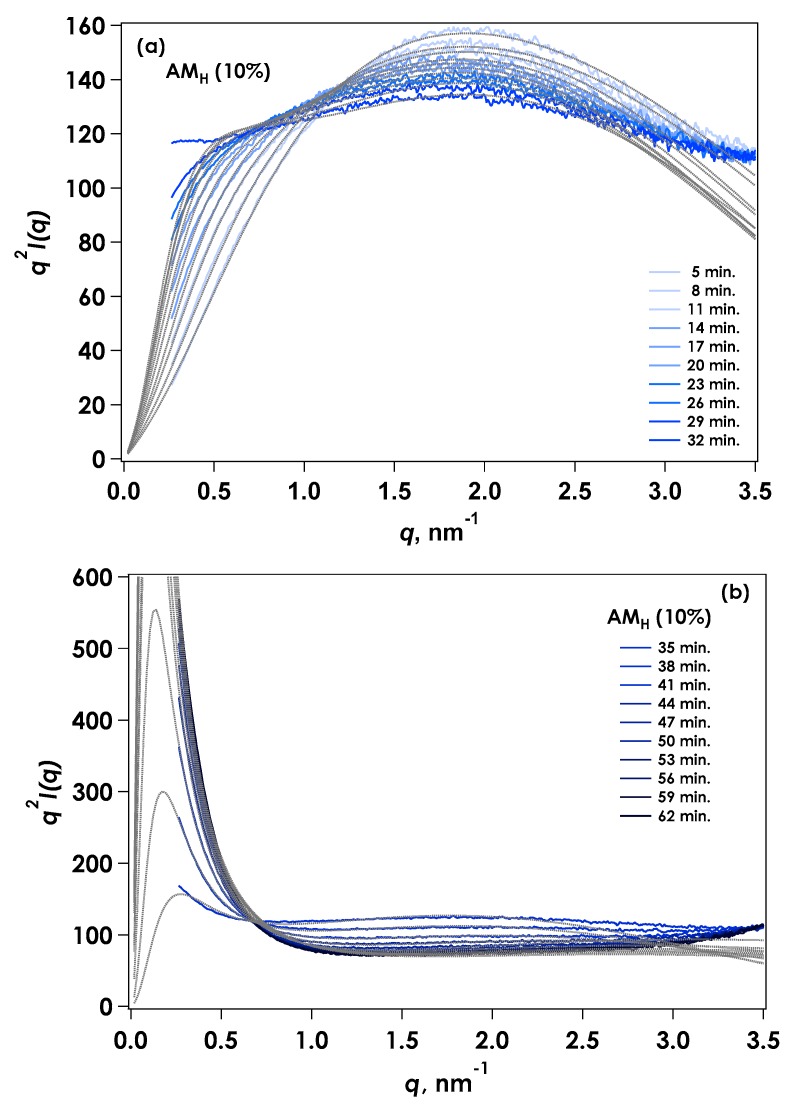
Kratky plots [*q*^2^*I*(*q*) vs. *q*] for SAXS from AM_H_. Dotted lines are fitted curves calculated from Equation (2). (**a**) Initial stage of reaction (5–32 min); (**b**) Final stage of reaction (35–62 min).

Figure 6 shows the Kratky plots for tr-SAXS obtained during monitoring of the gelation of AM_H_ by in situ neutralization. Compared with the AM_L_ system, the AM_H_ system contained a steeper slope increase in the smaller angle region, indicating greater aggregation. However, it appeared to have a trend similar to that of the AM_L_ system. Thus, the experimental results can be provided by the scattering function, including the Debye-Bueche model for the aggregated component, as:

(2) $$I\left(q\right)\sim \frac{1}{q}\cdot {\left[\frac{J_{1}\left(qR_{c}\right)}{qR_{c}}\right]}^{2}+\frac{I{\left(0\right)}_{DB}}{{\left(1+a^{2}q^{2}\right)}^{2}}$$

where *a* is a measure of the extent of inhomogeneity. The spatial distribution of the aggregated structure in the AM_H_ system was determined to be different from that for AM_L_ using two types of applied functions. Figure 7 represents the results of fitting with Equation (2). The calculated results agreed well with the experimental data. The inhomogeneous area appeared to spread as the molecular weight increased.

**Figure 8:**
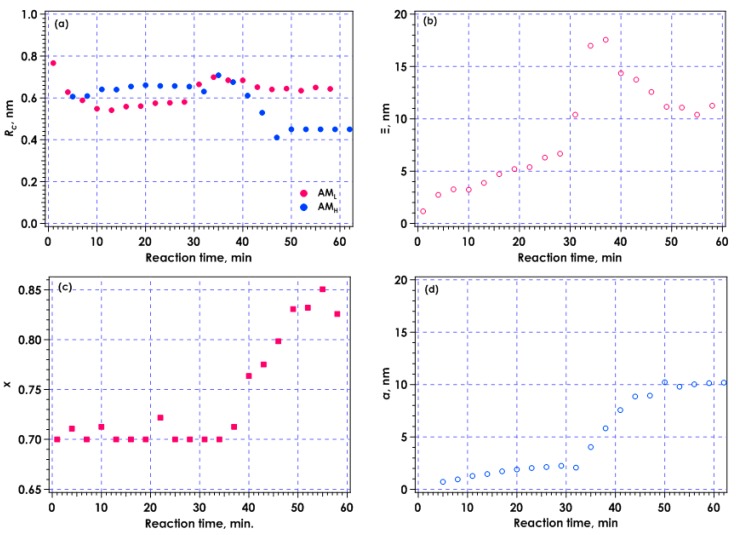
Time course for parameters evaluated from fitting with scattering functions. (**a**) Cross-sectional radius of rod, *R_c_*, for AM_L_ and AM_H_ system; (**b**) Correlation length *Ξ* for AM_L_; (**c**) Exponent, *x*, for AM_L_; (**d**) Measure of inhomogeneity, *a*, for AM_H_.

Figure 8 shows the time course for each parameter evaluated by curve fitting with Equations (1) and (2). Figure 8a represents the time variation in *R_c_*. The value of *R_c_* changed only slightly with reaction time, and was nearly constant at about 0.6 nm, corresponding to the radius of a single amylose chain. This result suggests the existence of dissolved amylose chains in the overall reaction process. The *Ξ* and *x* values were included in the exponential function of the aggregated component in the AM_L_ system. The value of *Ξ*, corresponding to the aggregation size, was several nm initially, then increased dramatically to about 17 nm at the gelation point, which occurred through formation of a cross-linking zone with aggregation and crystallization of amylose chains. The value of *Ξ* tended to decrease after gelation. The exponent *x* increased from 0.70 to about 0.85 after gelation. When the value of *x* was 2, a Guinier-type region was indicated, with a Gaussian density distribution. Thus, the aggregation structure had a wider size distribution immediately after gelation, then the aggregation restructured. In contrast, an AM_H_ system was analyzed using a Debye-Bueche-type function to evaluate *a* value, which was several nm and increased to about 10 nm after gelation. After 50 min, fitting was performed while maintaining an *R_c_* value of 0.45 nm to avoid the effect of the other component.

## 3. Conclusions

The gelation and structural formation of AM_L_ and AM_H_ dissolved in alkaline solution by in situ neutralization could be monitored with time-resolved small-angle X-ray scattering (tr-SAXS). A sharp increase in slope was observed for both types of amylase after gelation. This behavior was consistent with the occurrence of the gelation point. The results showed that the aggregation of amylose chains occurred at the gel point with crystal growth. The aggregation appears to function as a junction zone, with the rod-like amylose chains in the solution region promoting connections. As a result, the amylose chain can form a network structure.

## 4. Materials and Methods

### 4.1. Materials

Amylose samples with a low molecular weight, AM_L_ (*M*_w_ = 1.8 × 10^4^), was purchased from Hayashibara Co., Ltd. (Amylose EX-III, Okayama, Japan) and samples with a high molecular weight, AM_H_ (*M*_w_ = 2.2 × 10^5^), were synthesized using phosphorylase enzymes [18,19]. Other reagents were purchased from Wako Pure Chemical Industries, Ltd. (Osaka, Japan).

### 4.2. Gelation Method by In Situ Neutralization

Amylose samples were dissolved in 1 mol/L aqueous sodium hydroxide solution by mixing with a magnetic stirrer. In situ neutralization was conducted by addition of formamide to the sodium hydroxide aqueous solutions. This slow process is based on the chemical reaction that produces formic acid and ammonia. For gelation, 2 equivalents of formamide per 1 mol/L sodium hydroxide aqueous solution were added and mixed with a magnetic stirrer for 1 min to form a gel.

### 4.3. Viscosity Measurement

A rotational viscometer (Brookfield Co., Ltd., USA, RVDV-II + P CP) was used to obtain viscosity measurements. The amylose sol sample was added to the sample holder. Measurements were started after adding formamide at 20 °C and at 150 rpm rotational speed.

### 4.4. Small-Angle X-ray Scattering

Small-angle X-ray scattering (SAXS) measurements were obtained at the SPring-8 facility (BL-40B2) in Japan. The wavelength of the incident X-ray beam was 0.1 nm. The scattered X-rays were detected by an imaging plate placed about 1 m from the sample holder. The two-dimensional images obtained were transformed into one-dimensional data by circle averaging. Samples were mixed, followed by immediate insertion of the flat cell and placement in the sample holder, to start time-resolved measurements. The scattering data from solute was calculated as excess scattering by subtraction of scattering from solvent.
